# Crowding effect of institutional openness based on the big data algorithm on the efficiency of new energy technology innovation

**DOI:** 10.3389/fbioe.2023.1178737

**Published:** 2023-04-07

**Authors:** Ziying Cao, Leren Qian

**Affiliations:** ^1^ School of Economics Management and Law, Hubei Normal University, Huangshi, Hubei, China; ^2^ School of Computing and Augmented Intelligence, Arizona State University, Tempe, AZ, United States

**Keywords:** innovation efficiency, new energy technology, big data algorithm, institutional openness, development of the NEV industry

## Abstract

In recent years, new energy vehicles, as a high-tech industry, have developed rapidly. This paper uses “number of new energy project personnel” and “hours of R&D (research and development) personnel” as design indicators to evaluate the investment of innovative talents in enterprises. This paper first introduces the supporting factors of the innovation environment in the input of innovation resources, and conducts research from four perspectives: human resources, innovation R&D, technology acquisition, and environmental support. In the construction of the innovation output index system, this paper outlines that the technological innovation (TI) achievements of enterprises are related to factors such as technological capabilities, profits, and market competitiveness of enterprises. Finally, this paper evaluates it from three aspects: the research and development achievements, the economic benefits obtained and the competitive benefits of the enterprise. The results show that from 2018 to 2022, the average technological innovation efficiency of new energy enterprises is 1.06; TI’s efficiency indicators in the past five years are all above 1, and the overall improvement trend of TI is relatively stable. The new energy vehicle collaborative innovation system constructed in this paper will promote the overall development of the new energy vehicle industry.

## 1 Introduction

In recent years, the new energy vehicle (NEV) has been strongly supported by the state, and the NEV industry has developed rapidly. The new energy vehicle is a high-tech industry with high technology content, which requires a lot of capital and technology accumulation. Therefore, in the process of the development of the NEV, how to reasonably allocate innovative resources and improve economic efficiency is the key to the development of NEV. TI is the key link between economic transformation and industrial upgrading. However, due to the late development of NEV, the development of TI efficiency is still unclear. Therefore, in the NEV industry, the efficiency of TI is key to realizing the market development of NEV enterprises. Some advanced new energy technologies, such as automobiles and environmental protection need to be studied further.

In the big data (BD) environment, the strategic model of TI efficiency selected by Chinese enterprises has not only become a key factor in determining the development direction of enterprise innovation capability but also determines the survival and development of enterprises in the future. Hanif et al. (2020) aimed to assess the impact of human capital development on renewable and non-renewable energy consumption options. In addition, they also studied the depth of interaction effect of TI. Godil et al. (2021) believed that economic growth, TI, and renewable energy had a significant impact on CO_2_ emissions in China’s transportation sector. Caineng et al. (2021) believe that new energy will play a leading role in the third energy conversion and that it will dominate carbon neutrality in the future. Zhang et al. (2021) believe that low carbon energy TI is conducive to both environmental protection and economic development. Bettin (2020) put forward different concepts of TI and technological diffusion and integrated the methods discussed to revise the overall TI system method to study energy TI, such as energy storage. Pless et al. (2020) believed that clean energy innovation was key to decarbonization in the low-cost energy sector, and used a large amount of public research and development funds for energy innovation. Their research did not take into account the future planning of new energy technologies.

There is a significant correlation between the depth and breadth of technological diversification and the efficiency of enterprise innovation in the BD environment. Chen et al. (2018) systematically reviewed the typical innovation models and their shortcomings in the world. They proposed a new paradigm of overall innovation on the basis of learning from oriental wisdom and best innovation practices. Chege et al. (2020) believe that regulatory factors such as entrepreneurial innovation enhance the impact of TI on organizational performance. They examined the relationship between TI and enterprise performance in Kenya. Buglioni et al. (2021) believed that in the past decade, some TI have led to more repeatable, selective, and scalable photo-induced reactions. Muller et al. (2021) believed that TI often led to the redesign of the business models of established companies. They outline that acquiring, absorbing, transforming, and utilizing knowledge from the environment enables enterprises to participate in both exploratory and developmental innovation strategies. Borowski (2021) explores how innovation and new technologies enable companies to operate in a changing environment and discusses the importance of innovation strategies. Adkin (2019) believes that innovation is the core element of climate change policy in many jurisdictions. Innovation has simplified as technology has developed and is linked with market-driven priorities, which have adapted to the interests of major emitters in the energy sector. However, innovation, as a framework for the transformation of a post-carbon economy, was severely restricted and Boudet (2019) believes that the centrality of energy in economic, political, and social systems, and the widespread impact of energy choices on the natural world and public health, means that new technologies often stimulate public reactions. This review covers large-scale energy infrastructure projects such as utility scale wind and solar energy, fossil fuel mining, and marine renewable energy (Boudet, 2019), exploring the contribution of their research to the efficiency of new energy TI.

Based on theoretical analysis, this paper includes several key research ideas. First, this paper reviews the relevant theories and methods of China’s NEV TI and analyzes the technological development of China’s NEV enterprises. In its performance evaluation, we used data retrieval to establish a performance evaluation index system. The DEA (data envelopment analysis) method was used to make a static evaluation of innovation efficiency. The conclusion of this paper puts forward some policy suggestions for the development of NEV in China. Within the research scope of the paper, there were three companies with insufficient scientific and technological output. The value of insufficient scientific and technological output was 0.19, represented by Company B. The innovation of this article lies in using the design indicators of “the number of new energy project personnel” and “the working hours of research and development personnel” in evaluating the investment of innovative talents in enterprises and introducing the support factors of the innovation environment into the investment of innovative resources.

## 2 Methods for the prying effect of new energy technology innovation efficiency

### 2.1 TI efficiency

The efficiency of TI refers to the essence of TI. It refers to the source of TI, which is also the core content of TI. However, the technological progress of enterprises is a comprehensive effect of higher input and output. It is not only a complex system but also a microcosm of the entire socio-economic system. To fully explore the development process of the core technology of enterprises, during this period, the technological progress of enterprises is dominant, so it is necessary to carry out a longitudinal and systematic evaluation and comprehensive comparison. This paper analyzes various factors affecting economic development in order to obtain the economic benefits brought by technological progress.

TI efficiency is a method of measuring TI behavior, which can be used as a measure of a country’s TI capability. This requires a comparison of the comprehensive competitiveness of enterprises in order to formulate relevant economic policies. Because TI cannot produce commercial value by itself, certain measures must be taken to achieve the minimum investment and maximum effect.

**FIGURE 1:**
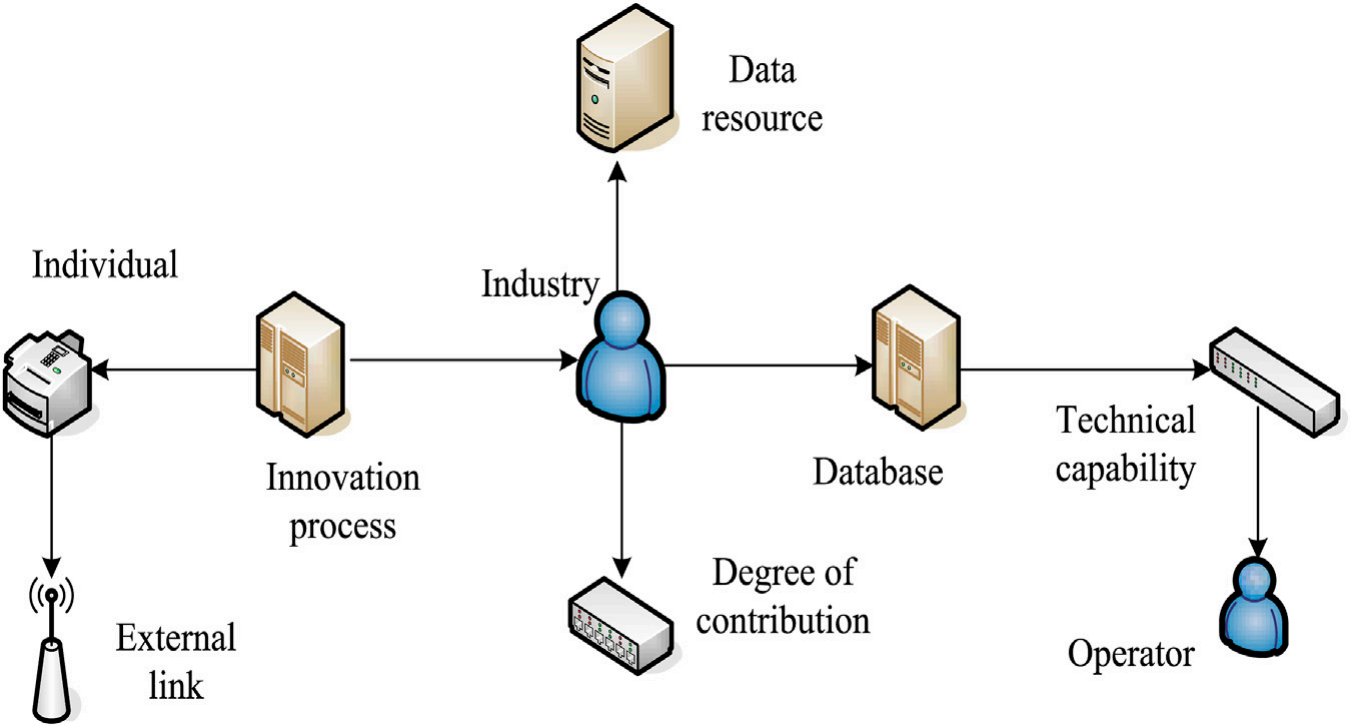
TI efficiency.

TI efficiency refers to the effective use of TI elements. When the output level of TI reaches a certain level, it is equivalent to saving investment in innovation resources. In the long term, the effectiveness of TI would directly affect the company’s sustainable development and market competitiveness. The efficiency of TI is shown in Figure 1. The extensive impact of new energy on the natural world and public health: with the vigorous development of the new energy industry, the bottleneck of low innovation level and low efficiency has gradually emerged. While capital is the first element of development and innovation, “financial leverage” has become the “transmission base” of the new energy industry capital. This can provide a “transmission base” of funds for the new energy industry and bring multi-directional and high-level value-added services to the new energy industry.

Renewable energy TI is an important driving force to promote the green transformation of the manufacturing industry (Saraswat et al., 2022). Data envelopment analysis (DEA) is the ratio of output to input $C_{T}$ :

$$C_{T}=\frac{Y_{j}\sum U}{\sum vx}=\frac{Y_{j}\left(U_{1}+U_{2}+\cdots +U_{i}\right)}{\left(v_{1}x_{1}+v_{2}x_{2}+\cdots +v_{n}x_{n}\right)}$$ (1)

The promotion of financial development and renewable energy utilization on environmental quality makes TI particularly important (Shah et al., 2021). Total factor productivity is calculated by using the Malmquist index. The Malmquist productivity index based on m and m+1 periods is

$$s_{z}\left(x^{m},y^{m},{x^{m+1},y}^{m+1}\right)=\frac{D_{0}^{m}\left({x^{m+1},y}^{m+1}\right)}{D_{0}^{m}\left(x^{m},y^{m}\right)}$$ (2)

$$s_{z+1}\left(x^{m},y^{m},{x^{m+1},y}^{m+1}\right)=\frac{D_{0}^{m+1}\left({x^{m+1},y}^{m+1}\right)}{D_{0}^{m+1}\left(x^{m},y^{m}\right)}$$ (3)

$D_{0}^{m}$ represents technological progress and change.

Institutional opening up is a new stage of China’s opening up, accelerating the transformation of government functions as its central work. From the perspective of institutional opening, the problems and causes of the transformation of the government functions of the free trade zone, this paper proposes the optimization countermeasures for the transformation of the government functions of the free trade zone in the context of institutional opening from the four dimensions of legal system construction, internal reform mechanism, administrative management system, and comprehensive supervision system, which can further improve China’s level of opening up.

The comprehensive productivity index $P_{z+1}\left(x^{m},y^{m},{x^{m+1},y}^{m+1}\right)$ is

$$P_{z+1}\left(x^{m},y^{m},{x^{m+1},y}^{m+1}\right)={\left(P_{m}\times P_{m+1}\right)}^{\frac{1}{2}}$$ (4)

In the formula, $P_{m}$ represents the technical efficiency change.

### 2.2 Index system construction of innovation efficiency

In the evaluation of innovation efficiency, the main contents are human resources, innovation funds, technology acquisition, and environmental support. The design of the innovation efficiency index is the basis for the evaluation of innovation efficiency. The following principles must be followed during the evaluation of innovation efficiency.

Reliability of indicator sources: this paper analyzed the relevant indicators of phase ratio innovation evaluation through sorting and summarizing some national documents. Previous research is based on previous theoretical and empirical research, which focuses on the reliability of theoretical maturity and empirical research, and includes indicators with high reliability and suitability for the research scope, thus improving the scientificity and rationality of the system.

The complete principle of the system: this paper takes the technical innovation efficiency of the NEV company as the research object, and its essence is the embodiment of the system operation efficiency. When designing indicators, people should not neglect the input and output of a certain aspect to avoid deviation.

The availability principle of indicator data: the statistical data of the new energy industry should be widely collected, with the annual report, development report, and the annual report published by the company as the main content, and the calculation of alternative indicators and original data should be used. This can eliminate design indicators that cannot be obtained to ensure the reliability and availability of indicator design.

**FIGURE 2:**
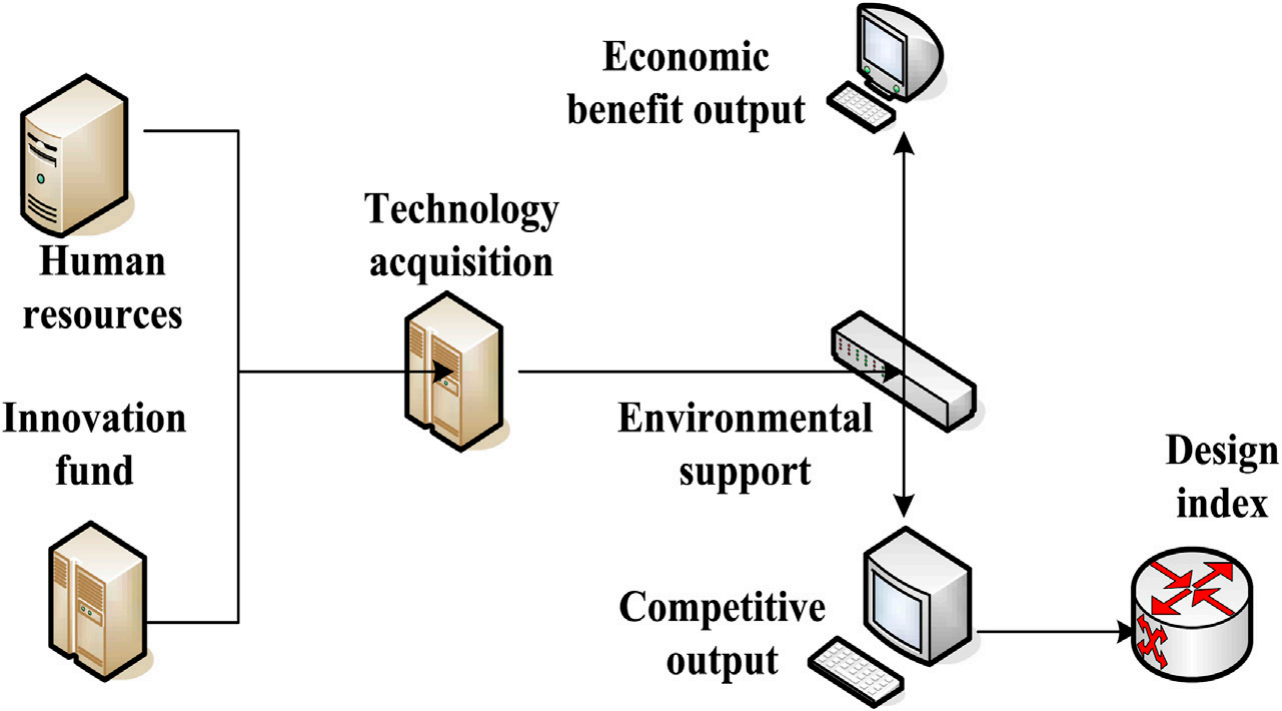
Index system construction of innovation efficiency.

The comparability principle of the same index: the concept, scope, mathematical processing method, and measurement unit of the index should be clearly defined. To avoid the subjective systematic error caused by individual differences, it should also reflect the real situation of the measurement data, thus making the research more repetitive. The index system construction of innovation efficiency is shown in Figure 2.

#### 2.2.1 Construction of the input indicator system

The existing research on the design of enterprise TI investment indicators is mostly constructed from the perspective of the human resource investment and R&D investment, which also needs to consider other factors.

##### 2.2.1.1 Human resource investment

Taking the new energy automobile industry as an example, this paper selects human resource investment as the evaluation index. In addition, in measuring the efficiency of TI, this paper refers to the concept of “full-time equivalent of R&D personnel” commonly used in other countries’ literature. Finally, this paper evaluates the investment of innovative talents in enterprises based on the design indicators of “number of new energy project personnel” and “R&D personnel hours”.

##### 2.2.1.2 Innovation and R&D investment

Based on the existing data and research results, this paper makes a detailed analysis of the investment projects of the TI fund. For example, “internal investment of R&D funds” is calculated according to the detailed items of the company’s actual financial expenditure. Vehicle enterprise expenses mainly include labor costs, raw material costs, and the purchase of fixed assets. This is a flow indicator reflecting the capital investment of an enterprise in a financial year. It reflects the capital investment intensity of a company, thus increasing an “R&D capital stock.” The stock index reflects the investment capacity of the enterprise’s innovation capital and how it is accumulated. At the target stage, the output of innovation is usually accumulated over a period of time. This paper believes that the innovation output is based on the existing stock, and this paper adopts the perpetual inventory method.

##### 2.2.1.3 Technology acquisition input

In addition to enterprises’ investment in TI, this can be obtained not only from themselves but also from the outside. China is a developing country. The development of the automobile industry is relatively lagging, and there is still a big gap between its technical level and that of the developed countries technical level. Therefore, the introduction of technology from developed countries is an important “catalyst” for enterprise TI. Therefore, based on “technology investment,” this article adds “external investment,” reflecting the company’s investment in procurement technology. “Contract amount of technology introduction from other countries” refers to the investment made by enterprises to introduce technologies from other countries, and since the capital introduction commitment of other countries is generally recorded in the form of contracts, the contract amount shall prevail in this paper.

##### 2.2.1.4 Environmental support investment

With charging facilities, maintenance point setting, warranty service, and other basic services for NEV, and the external environment as the control variable, the innovation efficiency of each sample company has improved overall. For this reason, this article first introduces the supporting factors of the innovation environment in the investment of innovation resources and studies them from the four perspectives of human resources, innovation research and development, technology acquisition, and environmental support.

#### 2.2.2 Construction of the innovation output indicator system

The TI achievements of enterprises are related to the technological capabilities, profits, market competitiveness, and other factors of enterprises, so this paper evaluates them in terms of three aspects: research and development achievements, economic benefits obtained, and competitive benefits of enterprises.

##### 2.2.2.1 Output of scientific and technological achievements

Scientific and technological achievements are the most direct manifestation of TI activities of enterprises. From the perspective of the availability of TI, this paper excludes a number of TIs, a number of technical documents, a number of technical innovation proposals, and other indicators of TI and adopts three indicators: “patent authorization number,” the “number of scientific and technological papers published,” and “technology market transaction volume.” These three indicators comprehensively describe the company’s TI output from the three dimensions of patent, literature, and market.

The patent database of the NEV is divided into two types, one is the number of applications, and the other is the number of licenses. Although the patent data are the innovation achievements of enterprises within one year, it would also change due to the number of reviewers.

This paper evaluates the “number of patent applications.” The number of scientific and technological papers published is also an important indicator of the TI capability of enterprises. Given the complexity of scientific and technological achievements, the “weight” of current patents varies greatly. Therefore, the statistics of the number of patents can only reflect a part of the existing knowledge reserves of enterprises, while the data of simply using patents would underestimate the unpatented technology of the company.

The trading volume of the technology market shows that one of the obvious shortcomings of the text statistics is that its form of expression is too simple and has nothing to do with the actual value. In order to fully reflect such innovative, scientific, and technological achievements with high application value, this paper selects the “technology market turnover” as an auxiliary indicator.

##### 2.2.2.2 Economic benefit output

The ultimate goal of enterprise innovation investment is to obtain economic benefits; that is, the ultimate achievement of the enterprises is to obtain commercial benefits. The main form of commercialized income is the business quality and profit of the enterprises. The sales revenue of new products reflects the direct economic benefits of its TI to the company, while the total profit of new products can reflect its promotion of quality. In order to fully reflect the economic value of the TI achievements of the NEV companies, this paper uses three indicators, namely, the total profit of new products, the added value of new products, and the sales income of new products, to measure the economic benefits and output of the NEV company.

##### 2.2.2.3 Competitiveness benefit output

The success of TI can improve the company’s market position, brand utility, externality priority, and other non-quantitative benefits. Its benefits are mainly reflected in two aspects: one is the labor productivity of the enterprise, and the other is the market share of the sales output value. In the process of TI, this paper selects two indicators, namely, “labor productivity” and “share of sales output value,” to measure the output capacity of TI.

Enterprises need to adjust and configure the resource structure of the organization, find appropriate technology diversification strategies according to their own technology and capability characteristics, and actively promote enterprise innovation to meet the needs of the BD era.

In this BD environment, the strategy of TI would directly affect the future development of the enterprises, the allocation of resources, and the formation of the innovation environment in the whole country. However, at the same time, increases in data collection costs and communication and coordination costs across industries, fields, and departments would also have a negative impact on the company’s innovation performance. In the context of BD, the ability of enterprises to obtain and process computing information is also improving. However, due to the characteristics of the enterprises, there are differences in the core technology capabilities of knowledge collection, coordination, and combination among enterprises and the use of BD. Enterprises should adopt appropriate technology diversification strategies according to their core technological capabilities. At the same time, the era of BD is not static. With rapid changes in technology and BD, the company’s dynamic capability has a great impact on the decision making of technology diversification. In the BD environment, enterprises must always pay attention to changes in the environment and rationally allocate their various knowledge resources to promote scientific and TI and achieve reasonable decision making.

The development of new energy is the key to adjusting the energy structure and establishing a clean and efficient energy system. The innovation of the new energy enterprises is the main starting point and entry point for the development of new energy. The interaction between market-oriented innovation and new energy enterprises is an important starting point to alleviate the difficulties of innovative financing of new energy enterprises. The development status of the new energy industry reflects the development level of China’s strategic emerging industries to a certain extent, which is conducive to the better service of the venture capital industry of the new energy industry, and helps China seize the commanding heights of the new energy industrial innovation as soon as possible. Starting from the concept of innovation efficiency of new energy companies, this paper defines the innovation efficiency of new energy companies, that is, the contribution of innovation input factors of new energy companies to their innovation output. It is difficult to directly observe innovation efficiency, which needs to be measured by a reasonable measurement method.

The expression of the DEA super efficiency model is as follows:

$$C_{X}=\min\left[\rho -\gamma \left(\sum s^{-}\left(1+s^{+}\right)\right)\right]$$ (5)

Innovation efficiency $C_{L}$ :

$$C_{L}=\beta^{2}+v\beta^{3}+{Size\beta }^{4}$$ (6)

$$k_{L}=\rho^{2}+v\rho^{3}+{\xi \rho }^{4}$$ (7)

β is a constant term, and ξ is a residual term.

## 3 Results of the prying effect of new energy technology innovation efficiency

The data collection period for this article was 2018–2022. On the whole, in 2018–2022, the average TI efficiency of new energy companies was 1.06, indicating that the TI efficiency of the whole industry was higher than that in 2018. Moreover, the indicators of TI efficiency in the past five years are all above 1, indicating that the overall trend of TI is relatively stable. This paper analyzes the reasons for the improvement of enterprise technical efficiency, and it can be seen that the change in pure technical efficiency has maintained a steady fluctuation in the past five years. In the past five years, the scale efficiency of NEV companies has decreased rather than increased. This shows that the scale benefit control of the enterprise would gradually become the bottleneck restricting the development of the company’s TI. Therefore, how to adjust the innovation investment scale of the company is particularly important. In recent years, China’s scientific and technological progress has shown a steady growth trend, which is closely related to the positive response of Chinese enterprises to national policies and the strengthening of cooperation among industry, university, and research. Technological progress is an important guarantee for TI. Compared with technology-intensive and knowledge-intensive industries, such as aerospace and bioelectronics, the technological advantages of the automobile industry are not obvious. The development history of the automobile industry in the world for a hundred years has proved that TI is the key factor for enterprises to grasp the technological life cycle. As a rising star, China attaches importance to knowledge storage, excavates the effectiveness of technology diffusion, and makes technological breakthroughs in the process of new energy transformation, which can be regarded as a strategic choice.

In particular, the pure technical efficiency of the company has improved year by year, and there is a growing trend. The improvement of pure technological efficiency is a manifestation of TI outflow, and its economic significance refers to the output growth brought by pure technological progress without changing the input factors. It can be seen that the NEV industry is a country with relatively late development, and it has strong late-developing advantages in technology research and development, management process, methods, and other aspects. Compared with pure technical efficiency, scale efficiency limits technical efficiency, indicating that the sample companies are in the scope of increasing returns to the scale on the whole. The scale of the company can be appropriately expanded and the existing industrial resources can be integrated, which can improve the scale efficiency of the company and further improve the technical efficiency of the company.

**FIGURE 3:**
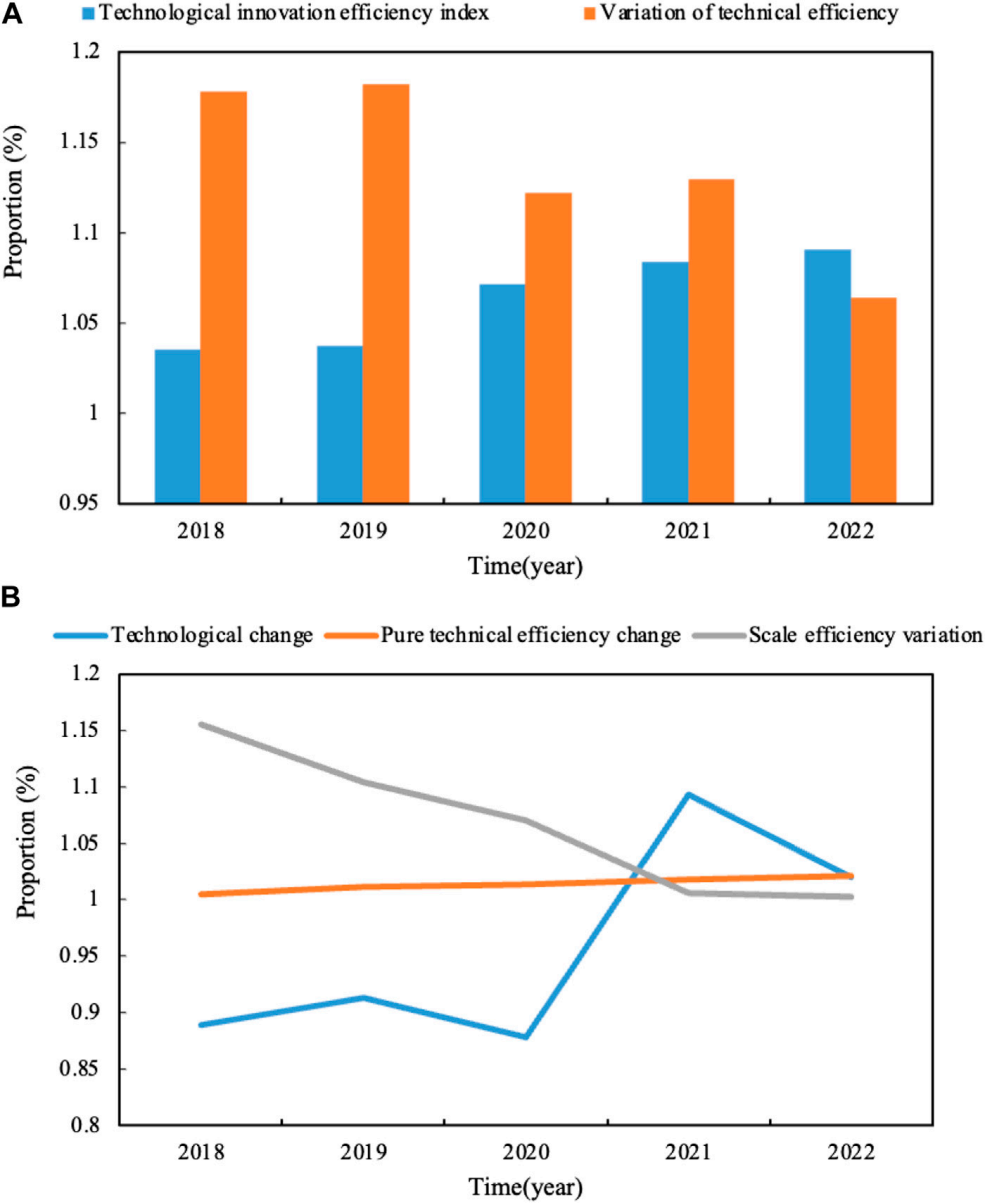
**(A)** Changes in TI efficiency index and technical efficiency. **(B)** Technology change, pure technical efficiency change and scale efficiency change.

There is a relationship of mutual support and coordinated development between the company, related companies, and supporting companies in the industrial chain. Their interests are mutually reinforcing, and the strong competitiveness of the companies would also promote the development of other members of the entire industrial chain. The new energy automobile industry is a highly related industry. In the process of development and production, it would consume a lot of resources, such as chemical, electromechanical, electronics, and materials*.* The strength of new energy automobile companies is reflected in the development of the new energy automobile industry, which can drive the development of other industries. At the same time, the development of the new energy automobile industry would also promote its development. The dynamic analysis of innovation efficiency of new energy automobile enterprises is shown in Figure 3 (the change of the TI efficiency index and technological efficiency is shown in Figure 3 a. Technical change, pure technical efficiency change, and scale efficiency change are shown in Figure 3 b).

New energy vehicle companies have implemented diversified development strategies. While developing pure electric vehicles, renewable energy vehicles, and hybrid electric vehicles, the company should also strengthen the combination with new technologies, such as the Internet and BD, and realize the development of the company by operating a variety of new products. While developing the diversification of the NEV, it should pay attention to the brand construction of the NEV, establish brand awareness, and develop brand products of NEV. At present, the brand technology in other countries is relatively mature and has a wide range of applications. It is difficult for Chinese enterprises to catch up with other countries. The Chinese NEV can assess the situation, give full play to local resources, and cultivate local enterprises of the NEV. Enterprises should seize the opportunity, seize the market, and constantly develop new products according to the market needs of some countries to increase the economic benefits of the enterprises.

Within the scope of the study, there are three companies with insufficient scientific and technological output, represented by Company B, and the value of the insufficient scientific and technological output is 0.19.

According to the specific situation of the company, Company C established a NEV research and development organization in 2018. At present, it has completed three electric vehicles and two hybrid electric vehicles, applied for 40 patents, and established a cooperative relationship with strong technology enterprises. For example, there are electric drive limited companies in the direction of electric motors and partners of China Automobile Research Institute. However, due to the company’s development strategy and the imperfection of relevant equipment, Company C plans to put a large number of production and sales in the world before 2020. The same is true for some auto companies today. Their economic situation is very bad, but their research results are quite effective. With the comprehensive development of the new energy industry and the continuous improvement of its supporting facilities, it would create a good platform for its economic value creation.

**FIGURE 4:**
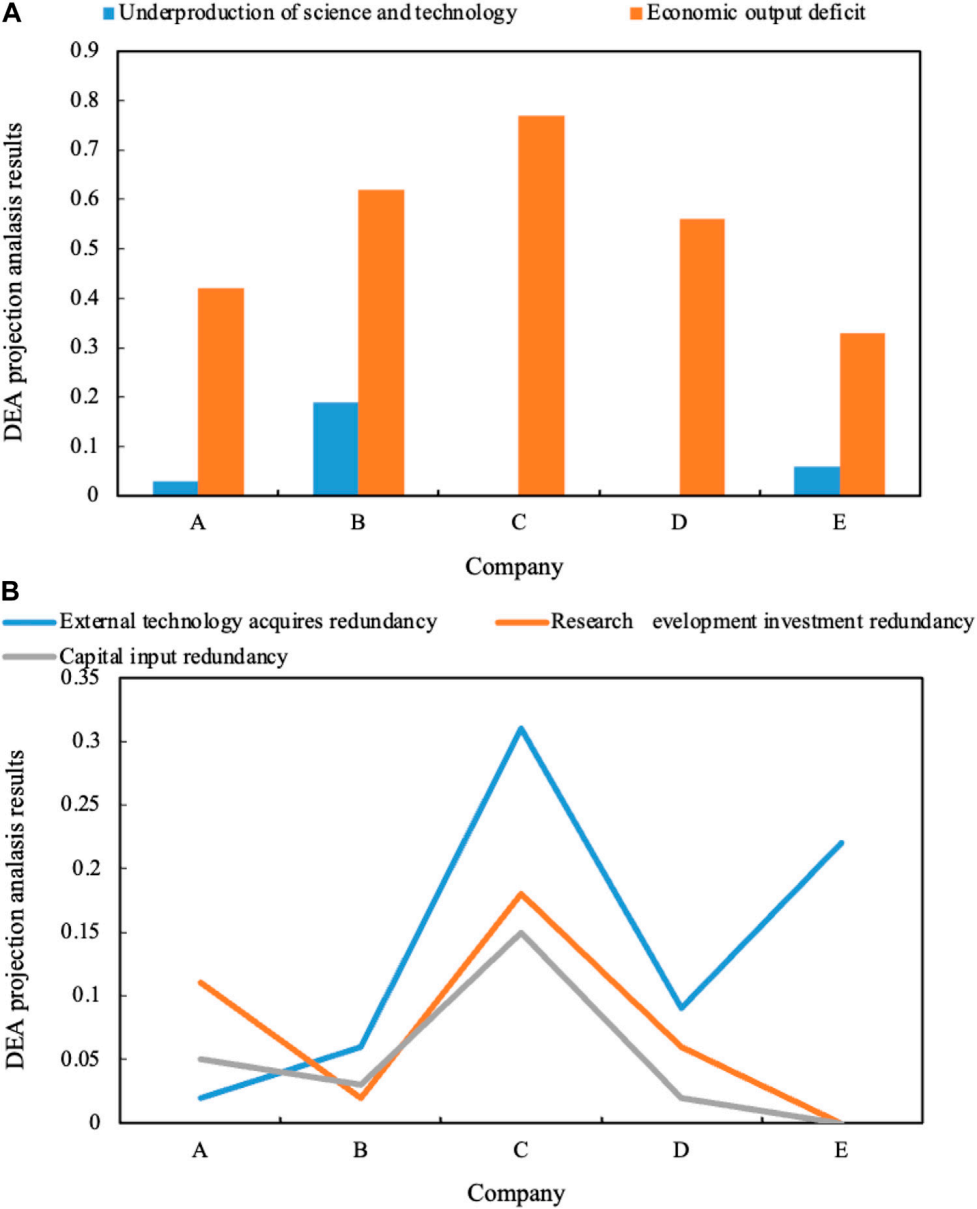
**(A)** Insufficient scientific and technological output and insufficient economic output. **(B)** External technology acquisition redundancy, R&D investment redundancy and capital investment redundancy.

The DEA projection analysis results of innovation investment of new energy automobile enterprises are shown in Figure 4 (insufficient scientific and technological output and insufficient economic output are shown in Figure 4 a. External technology acquisition redundancy, R&D investment redundancy, and capital investment redundancy are shown in Figure 4 b).

Today, the rapid development of science and technology, economic globalization, increasingly diversified consumer demand, high uncertainty of the market environment, and fierce competition among enterprises have broken the pattern of closed innovation of a single enterprise and formed a diversified, complex, and dynamic network structure. The enterprise constantly establishes a complementary innovation system with scientific research institutions, suppliers, government departments, and other stakeholders. The TI of the NEV is open, complex, and sustainable and includes batteries, motors, and power systems. These technologies and resources have been increasingly applied to the NEV industry. Because they involve too many disciplines, are too difficult, and are too complex, which are not available in the traditional automobile industry and cannot be achieved by a single company, in the NEV industry, how to integrate various scientific and technological resources and establish an open collaborative innovation model has become the direction of joint efforts of NEV manufacturers in all countries.

While improving the technical level, new energy automobile companies are also actively seeking cooperation with leading enterprises in the same industry to achieve the purpose of enhancing competitiveness. Collaborative innovation requires participating enterprises to have advantages in a certain field and participate in research through complementary advantages and joint investment. Collaborative innovation can bring many benefits, such as reducing repeated research investment and reducing the risk of research failure.

For a long time, the development level and manufacturing level of Chinese automobile enterprises have been relatively backward, and some other countries have imposed a technical blockade on Chinese companies. China adopted the strategy of “market for technology,” but failed to obtain the core technology, and the joint venture companies exclusively owned by others countries’ investors also failed to promote the overall technological upgrading of China’s automobile industry. However, in the field of NEV, there are currently positive restrictions, and Chinese enterprises can cooperate and develop together on an equal basis.

**FIGURE 5:**
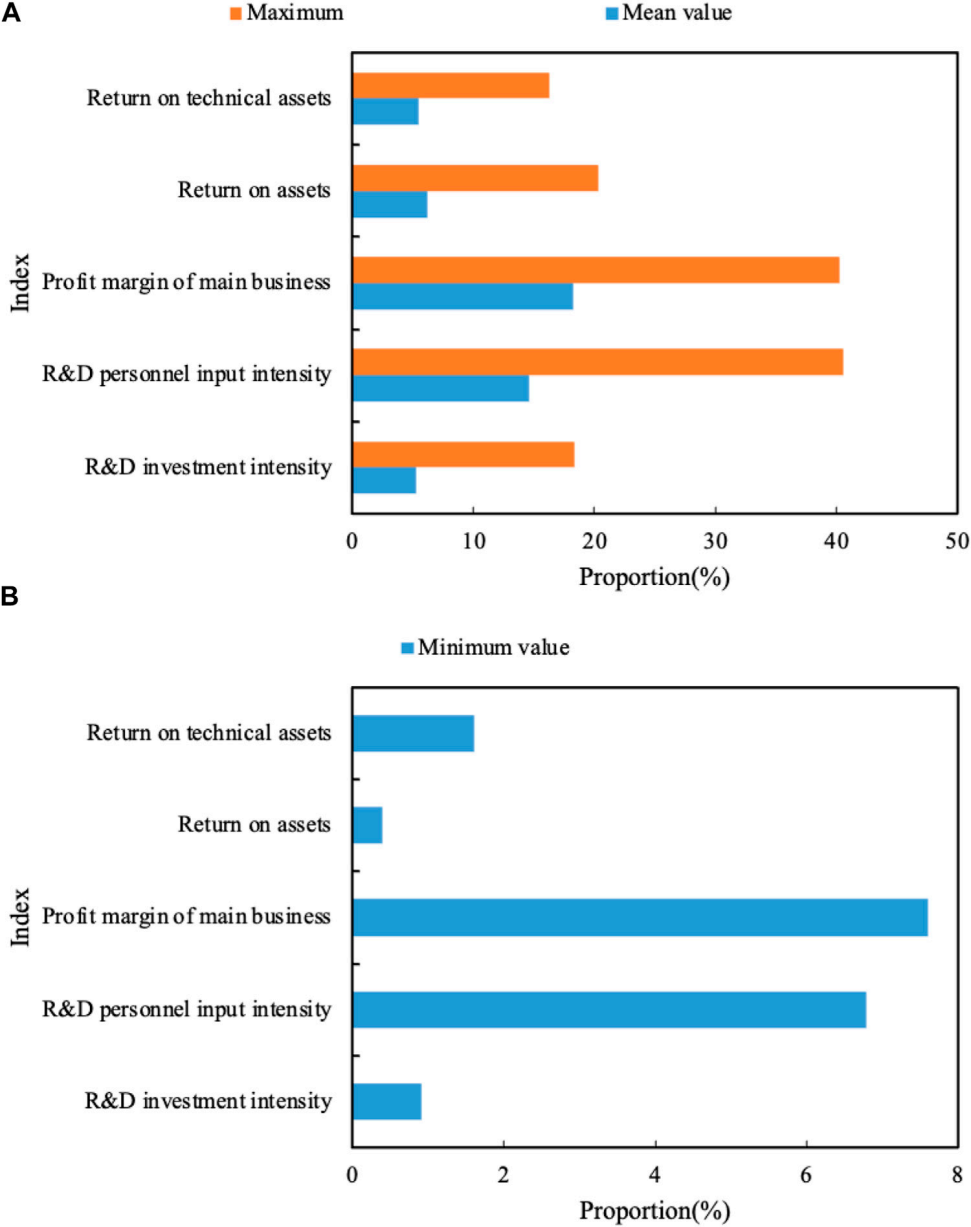
**(A)** Mean and maximum values of input-output indicators. **(B)** Minimum values of input–output indicators.

The descriptive statistical results of input–output indicators are shown in Figure 5 (the mean and maximum values are shown in Figure 5 a, and the minimum values are shown in Figure 5 b). The average investment intensity of R&D funds is 5.3%. The return on technical assets is 5.5%. There are differences in investment and output between companies.

In today’s rapid economic development, innovation has played an important role in promoting the development of human society. As a representative strategic emerging industry, new energy plays an important role in promoting economic, social, and long-term development. At present, China’s new energy industry has initially formed a scale, but the overall scale is small, the power support is not strong, and the level of innovation is not high. It needs to improve the research and development and innovative design capabilities of key core technologies in the new energy industry, and improve the innovation efficiency of new energy companies. This can cultivate new energy core technology and seize the commanding heights of the new energy industry and enterprise innovation. Because the innovation of new energy enterprises requires a large amount of capital, and the high cost and high risk make it difficult for the traditional financing mode to fill the capital gap of the new energy industry, and the capital problem has become the biggest bottleneck restricting the innovation of new energy enterprises.

The rapid development of the Internet of Things, Internet, cloud computing, blockchain, and other technologies has greatly improved the ability of BD analysis, processing, storage, and other aspects. This can promote the BD technology system with open source as the leading factor and multiple technologies and architectures coexisting. However, compared with some other countries, China’s current computer platform, distributed computing system, and BD presentation method cannot adapt well to the application of BD in various fields. At the same time, because enterprises are faced with real-time and dynamic data completely different from traditional data, BD processing tools need to have the technical ability to analyze BD in real time. This can solve the problems of new energy technologies in a timely manner and reduce costs.

**FIGURE 6:**
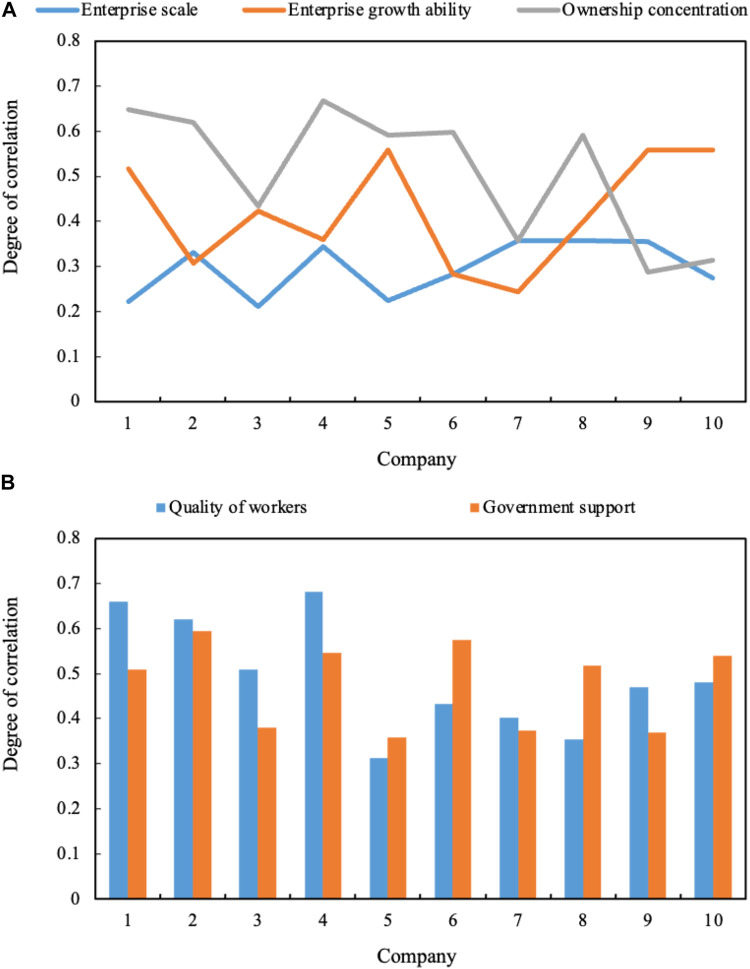
**(A)** Factors of firm size, firm growth capability and ownership concentration. **(B)** Factors of labor quality and government support.

The correlation degree of various factors of innovation efficiency is shown in Figure 6 (enterprise scale, enterprise growth ability, and equity concentration are shown in Figure 6 a. The quality of workers and government support are shown in Figure 6 b). The correlation degree of equity concentration is 0.51, and the enterprise scale is the lowest, 0.30.

Traditional technology finance focuses on the ends while emerging technology finance focuses on the means. Both traditional and emerging technology finance emphasize their essence of alleviating the pressure of small- and medium-sized technology financing and promoting enterprise progress. It has both the attributes of public finance and the characteristics of commercial finance.

## 4 Conclusion

This paper took the efficiency of TI of the NEV and its influencing factors as the main research object. Through discussion of research by scholars in a number of countries, we established the measurement index system of the efficiency of TI of NEV and evaluated the efficiency of TI of enterprises from the perspective of TI. There were many influencing factors of TI. Questions about how to select the appropriate influencing factors and measure their impact are very complex. If the new energy automobile industry wants to stand firm in future development, it must pass TI, improve innovation efficiency, and accelerate the upgrading of the industry. This paper put forward some countermeasures and suggestions for the development of the new energy automobile industry, hoping to play a certain reference role for the development of the new energy automobile industry. Due to the difficulty of collecting data on the TI of NEV companies and the limited level of theoretical knowledge, research on TI efficiency is not comprehensive. At the same time, it is also expected that the advantages of BD would have a certain impact on the TI efficiency of the NEV, thus providing a certain theoretical basis for the development of the NEV industry. This article contributes to the development of the new energy industry, exploring the level of innovation, and the efficiency of innovation. In the future, it will move forward to multi-directional, high-level value-added services.

## Data Availability

The original contributions presented in the study are included in the article/Supplementary Material, further inquiries can be directed to the corresponding author.
